# Interdisciplinary Approach to Spatiotemporal Population Dynamics: The North Orkney Population History Project

**Published:** 2019

**Authors:** Julia A. Jennings, Corey S. Sparks, Timothy Murtha

**Affiliations:** University at Albany – State University of New York; The University of Texas at San Antonio; University of Florida

**Keywords:** Historical Demography, Landscape, Households, Land Use, Historical Archaeology, Ethnography

## Abstract

The North Orkney Population History Project is a multidisciplinary data collection, digitization, and analysis effort that aims to reconstruct longitudinal demographic, environmental, and economic change. We describe the motivation, methodological approach, data sources, and some initial findings of the project. Detailed contextual information about a single community allows for the joint analysis of the changing population and changing landscape. The combination of diverse data sources and disciplinary approaches has resulted in findings that would not have been possible if each source had been considered in isolation. The approach adopted by the project offers a way to examine the interaction of a population with its landscape over a period of change.

## 1 PROJECT AIMS AND OVERVIEW

**Figure 1:**
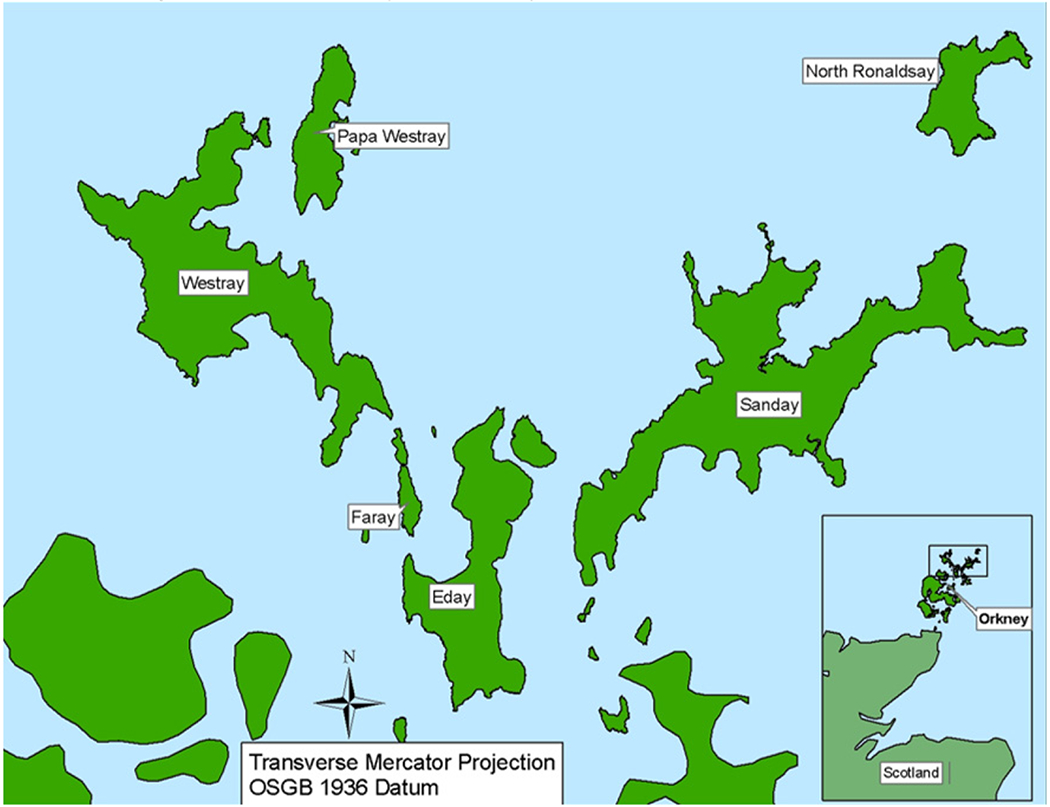
Map of North Orkney. The study area includes the six identified islands

**Figure 2:**
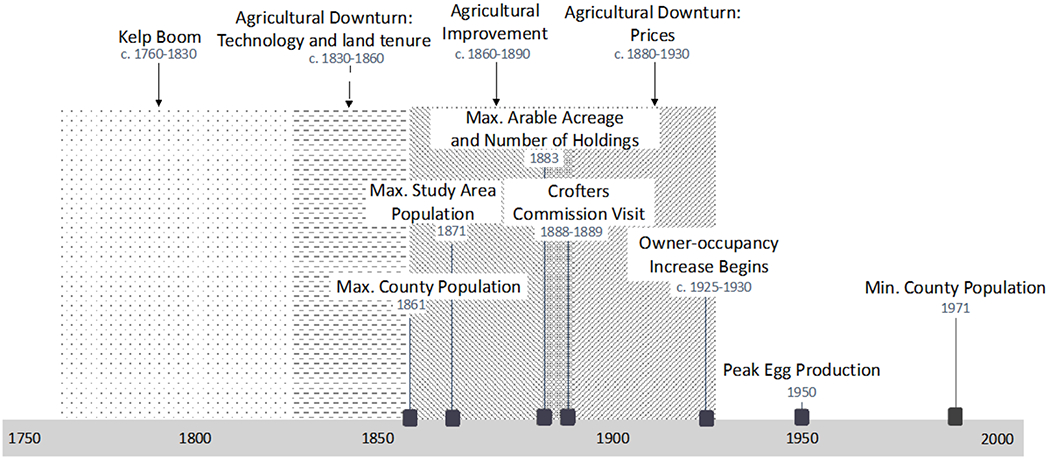
Timeline of selected events in North Orkney

The North Orkney Population History Project (NOPH) was designed to study change in population, economy, settlement, and land use in six of the northernmost Orkney Islands (Westray, Sanday, Papa Westray, Eday, North Ronaldsay, and Faray), located off the northern coast of Scotland (Figure 1). The study period (c. 1735-2000) covers the transition from a high fertility and high mortality demographic regime to one with low fertility, low mortality, substantial out-migration, population loss, and population aging. Economically, this period witnessed the shift from traditional subsistence agriculture and fishing to a modern system of livestock production for external markets. Figure 2 illustrates the timing of some of the important events and transitions in North Orkney. The NOPH investigates the links among these changes using an interdisciplinary set of approaches including historical demography, historical archaeology, ethnography, and spatial analysis. These complementary mixed approaches aim to corroborate, elaborate, and clarify findings of closely related research questions that address population and landscape processes in this setting (Mathison, 1988; Tashakkori & Teddlie, 1998).

The project is motivated in part by the lack of generalizability of studies of long-term population change dominated by the demographic transition theory (Davis, 1963; Notestein, 1945). Some critiques of demographic transition theory arise from the observation of diverse trajectories of population change (Aghajanian, 1991; Caldwell, Orubuloye, & Caldwell, 1992; Chesnais, 1992; Cleland, 1994) and others emphasize weak connections between demographic transition theory and the underlying mechanisms of population change (Mason, 1997; Szreter, 1993; Wilson & Airey, 1999). One response to these issues is to call for the collection and analysis of detailed demographic micro-data (Caldwell et al., 1992; Greenhalgh, 1995; Kertzer & Fricke, 1997; Szreter, 1996), and when possible, make comparisons across societies that have similar systems of data recording (Bengtsson, Campbell, & Lee, 2004; Lundh & Kurosu, 2014; Tsuya, Feng, Alter, & Lee, 2010). Yet, demographic micro-studies in isolation are insufficient to address general long-term population trends, which may take several generations to develop. To understand how a preindustrial demographic and ecological regime was altered and what kind of regime took its place, longitudinal population data are needed from a region with continuous settlement from preindustrial times until the onset of the modern regime. Longitudinal contextual information on the local environment and economy are also required for change to be explained. These kinds of contextual information are becoming increasingly common in historical demographic studies (Coughlan & Gragson, 2016; Hedefalk, Harrie, & Svensson, 2014; Sylvester, Leonard, & Gutmann, 2006), but it was not a traditional focus of the field. This type of investigation requires that the requisite documentary evidence have been persevered. Further, the application of additional methods from ethnology, archaeology, and environmental science supplement and aid in interpretation of demographic trends reconstructed from historical micro-data. Ideally, these forms of evidence should also be spatial, as demographic regimes have both temporal and spatial components and populations depend on necessary resources that are distributed across a landscape.

**Figure 3:**
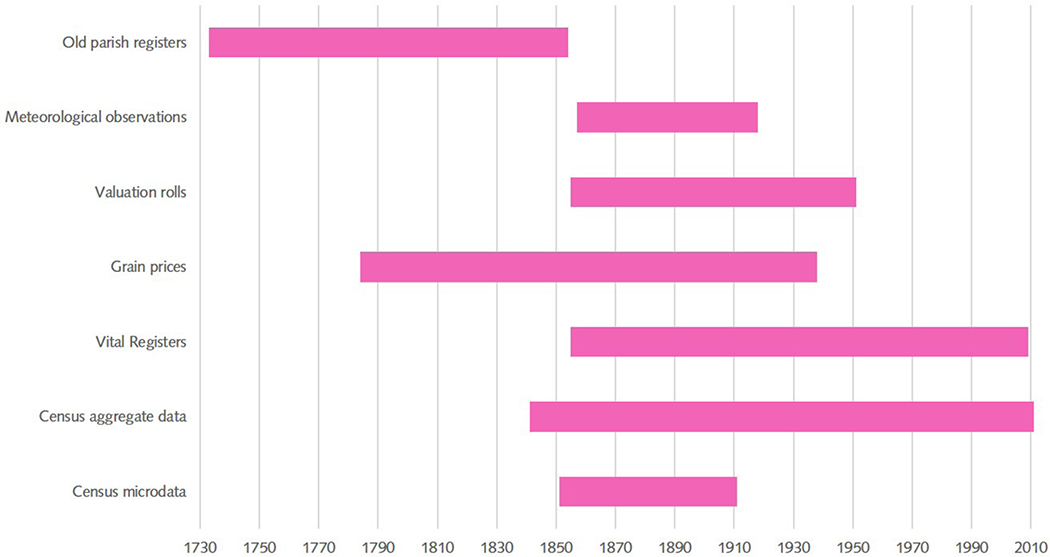
Temporal coverage of selected source material of the NOPH project

The NOPH aims to address these needs through a combination of a detailed study of a specific population, long-term historical evidence on the same population, and longitudinal landscape data (Figure 3). The northern Orkney Islands have been continuously settled since the Norse era (c. 850), and over 90 percent of modern inhabitants have surnames that are known in Orkney since the medieval period (Lamb, 2003). Old parish records date to the 1730s, but preservation and coverage is uneven. Civil registers (1855-2009) and decennial censuses (1851-1911) are available from the mid-1800s. Additional documentary material, including rentals, cadastral maps, and tax valuations, are available for reconstructing change in material conditions over the last 200 years. When compared with the rest of Britain, agricultural modernization and change began late in Northern Orkney, as owner-occupancy and the widespread mechanization of agriculture did not begin until the 1930s. The study area reached its peak population in the 1860s and 1870s, and this loss of population continued until the 1970s. This population loss has left a rich historical archaeological record of houses, farmsteads, field systems, dykes, drainage systems, and evidence of rural industries. Continuity of settlement ensures that the present population can provide insight into the processes of change that occurred within living memory or are part of oral history accounts. Data from Greenland ice cores (Alley, 2000) and time series of instrumental meteorological observations allow for the reconstruction of fluctuations in climate. Existing environmental data, including daily rainfall and temperature, soils, drainage, satellite imagery, and aerial photography are available, and the project conducted archaeological surveys of house lots, field systems, and grazing areas to study environmental change.

## 2 STUDY POPULATION

The six islands chosen for study form a cluster at about 59° N 3° W, the meeting of the North Sea and North Atlantic (Figure 1). Approximately 1300 people live on the islands today, while the maximum population was 6062 in 1861. Settlement is rural and dispersed, with only a few clusters, traditionally called townships, with the more populous islands having one or two small villages with accommodations and public houses. Farm structures have been rebuilt over time, but the majority of modern farms are located on named farm sites that date to before the eighteenth century, with some of even deeper origin (Marwick, 1952; Scott, Stevenson, & Stout, 2003). Families and individuals have moved about the landscape, but the farmsteads have been stable, providing a spatial point of reference. Farmstead names were regularly used in documents including the census, vital registers, maps, and rentals, and they can be used to trace the movement of people across the landscape.

Traditional agricultural production was organized to meet household subsistence needs and pay rents, often in kind. The system of production relied on a balance of arable grain production, predominantly oats and barley, and livestock raising, mostly cattle, sheep and poultry. Vegetables for household consumption were grown in small plots or household gardens. With limited land on the islands, the balance of production was critical, as grazing competed with arable land while providing important enrichment of arable land from animal manures (Dodgshon, 1994). Historical and archaeological evidence indicate that this system had changed very little since the medieval period (Fenton, 1997; Thomson, 2008a).

Fishing was a secondary sector of the islands’ economy. Many farmers owned small boats and access to the shoreline was guaranteed by law and custom. Farming families could supplement their production with fishing, while some households relied solely on inputs from fishing and related trades, such as fish curing and processing. Participation in fishing trips could explain a portion of the sex imbalance among young and middle-aged adults observed in 19^th^ century censuses, as some men would be away from home on seasonal fishing trips. Unlike Shetland to the north, the fishing trade in Orkney was less specialized and most who worked in fishing did so part-time. Fenton (1997) estimates that in the 1930s, 80% of Orkney’s fishermen were part-time workers who also had small agricultural holdings. The fish trade also attracted young, unmarried women to Orkney to work in fish processing in the late 19^th^ and early 20^th^ centuries (Fenton, 1997). However, these large processing plants were not located in the study area, so these workers are not observed in this dataset.

In the eighteenth century, a series of changes had dramatic effects on the northern islands. Individual-level demographic data from this period is sparse, and accounts of these changes rely on other sources. A kelp boom (c. 1780-1830) occurred, increasing the demand for labor to collect and burn seaweed to produce alkali for British industrial production of glass, soap, and dye. The population of the County nearly doubled in response (Thomson, 1983; 2008b). Much of this increase came from in-migration, although some changes in fertility and mortality may have also occurred (Brennan, 1979; 1983). The kelp bubble burst in 1830 when high government tariffs on imported sources of alkali were repealed, leading to a price collapse in Orkney kelp and a period of economic stagnation, reflecting a shift away from agricultural innovation and improvement during the height of the kelp boom (Thomson, 2008a). A period of low returns on labor associated with population pressure, limited access to markets, and few marketable exports followed the collapse of the kelp market in the 1830s and 1840s (Thomson, 1983; 2008b). This downturn coincided with the onset of a period of depopulation and out-migration, mostly to mainland Scotland, North America, Australia, and New Zealand.

**Figure 4:**
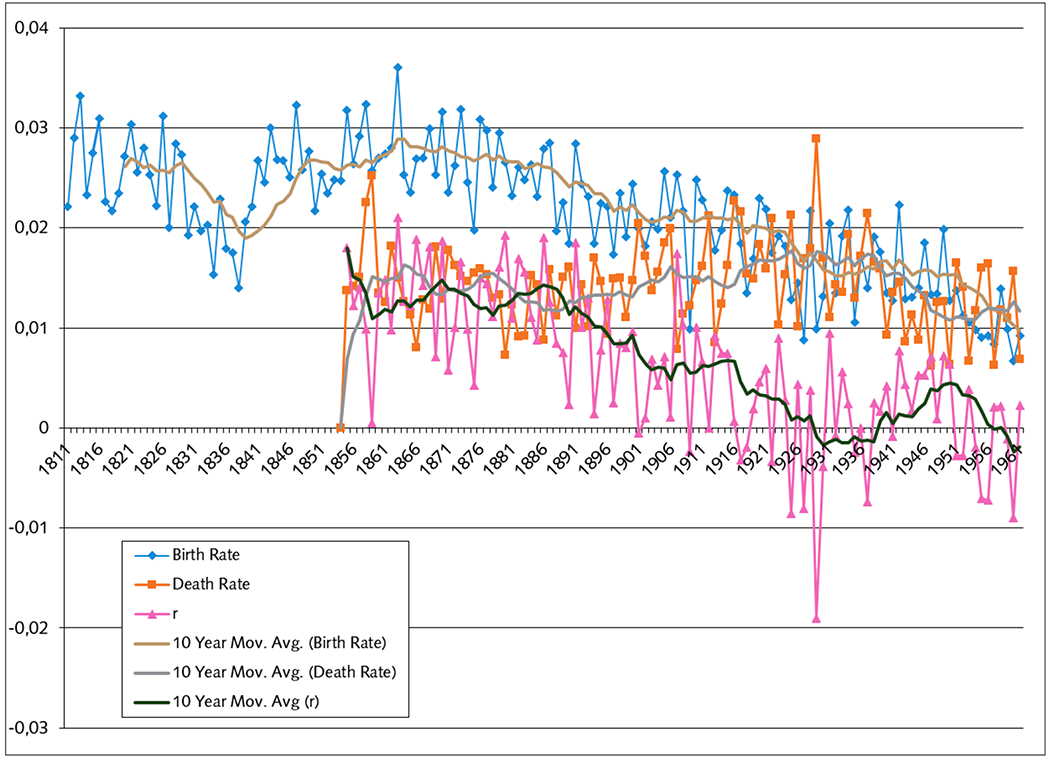
Time series of crude birth rates, crude death rates, and birth rate minus death rate (r), Westray, Orkney. Trend lines represent 10 year moving averages Sources: Old Parish Registers (pre-1855) and vital registers (1855-1961).

Agricultural improvement reached Orkney in the mid- to late-nineteenth century (Figure 2). Fields were reorganized, common pasture was enclosed, and other innovations, including new field drainage techniques and liming were adopted. These changes increased the amount of arable land under cultivation and the production of grain and other important crops, such as potatoes, increased accordingly. Steam shipping reduced the cost of exporting goods to market and encouraged the intensification of agricultural production (Schrank & Lockhart, 1995). Out-migration slowed but did not cease, likely because improved agriculture allowed greater production with fewer labor inputs. The period of expansion continued until the 1880s, when an agricultural downturn associated with competition from North America and Australia began (Thomson, 2008a). This agricultural depression led to additional out-migration (Pooley & Turnbull, 1998). The fertility transition began around the same time, which also contributed to population loss in the late 19^th^ century, as sustained mortality decline lagged behind fertility decline in the Northern Islands. Figure 4 shows crude rates, but age-specific and standardized rates indicate a similar pattern of relative fertility and mortality change (Sparks, 2007).

**Figure 5:**
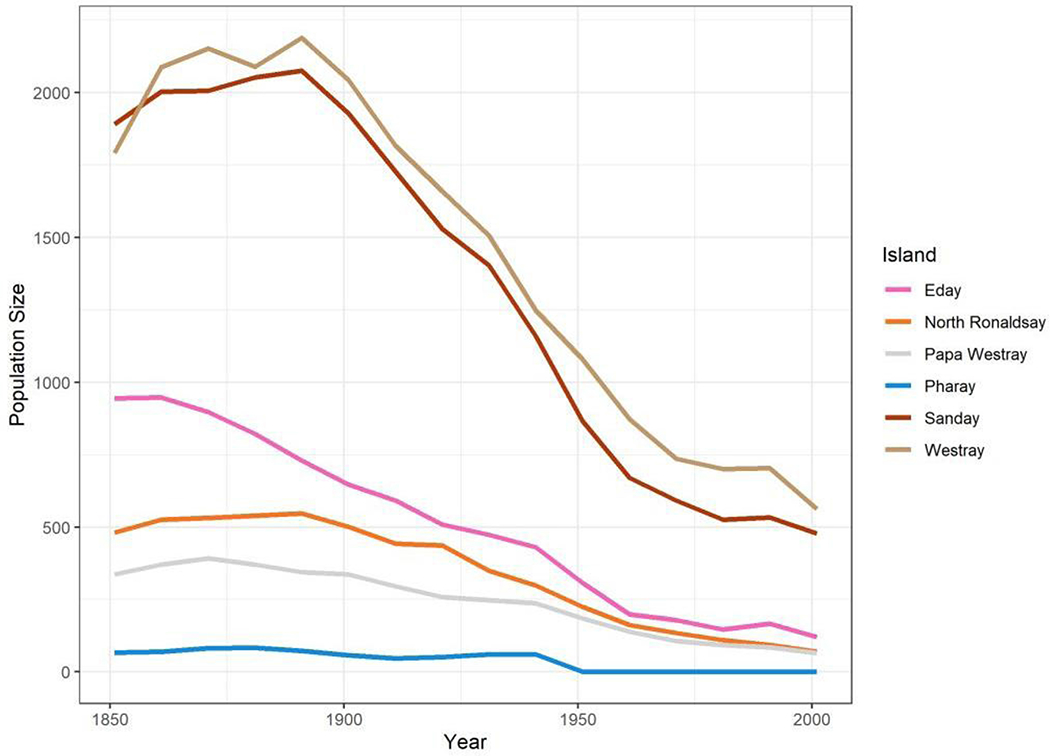
North Orkney census population sizes, 1851-2001

**Figure 6:**
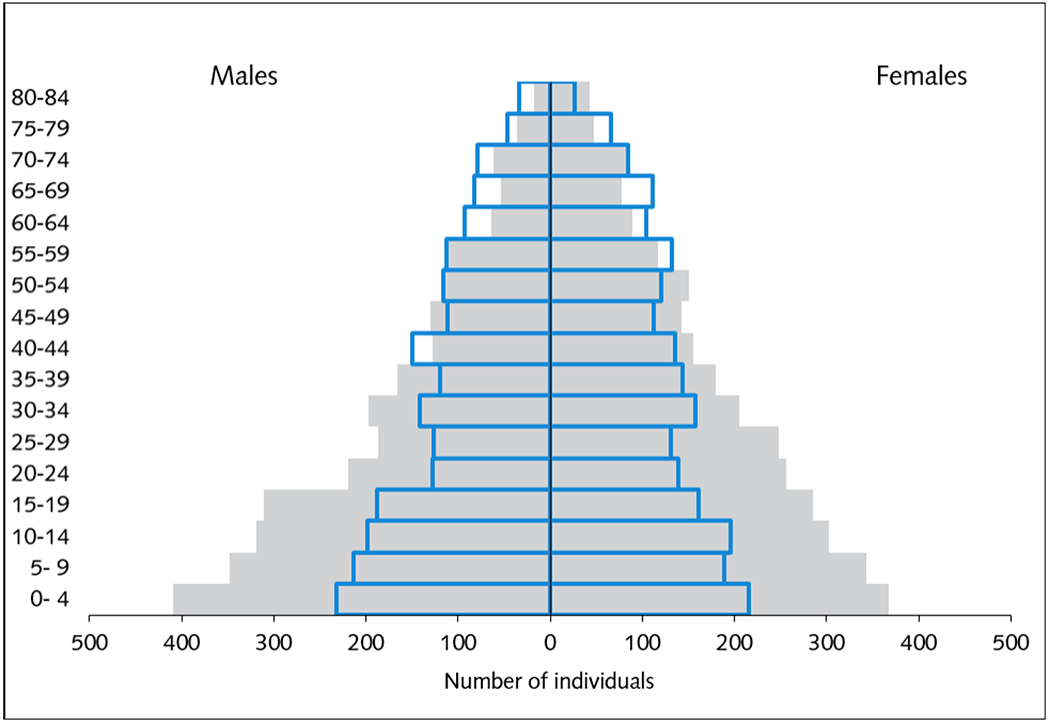
Sex and age structure of the Northern Orkney Islands, 1851 census (shaded) and 1911 census (outlined)

Ultimately, these changes represent a failure of demographic homeostasis, if it had existed in the preindustrial period. The population of the northern islands reached its maximum in 1861 and has declined ever since (Figure 5). The smallest island in the study, Faray, was completely abandoned in the 1950s. This trend of population loss and accompanying population aging (Figure 6) has occurred throughout the highlands and islands of Scotland, and in-migration has offset it in only limited places (Flinn et al., 1977; Hunter, 1991). One of the goals of the NOPH is to reconstruct and interpret the entire transition from the pre-industrial population regime to continued population decrease.

## 3 DATA SOURCES AND COLLECTION

The NOPH collected and digitized a diverse set of information on the study area. As some of these data sources are uncommon in historical longitudinal studies or are specific to the region, we describe the sources, method of collection, and post-collection processing and analysis. A timeline (Figure 3) illustrates the temporal coverage of some key sources.

The project personnel have changed from the inception of the study to the present. It was formerly a defined group of principal investigators, collaborators, PhD students, and undergraduate researchers, and it is now a group of scholars with current or former involvement in the project and ongoing interest in research and maintenance of the data infrastructure. This interdisciplinary group has included biological and cultural anthropologists, archaeologists, demographers, and specialists in spatial analysis. Data collection began in 2003 and was completed in 2019. Data linkage, cleaning, and coding are ongoing, as are analyses and research projects. Data products will be maintained for future analysis, and deidentified census data will be made publicly available for the research community. Other data sources cannot be made public in accordance with data access agreements with the General Register Office for Scotland, now known as National Records of Scotland, but may be made available for specific research purposes, including those not discussed in the original project aims, or collaborative projects.

### 3.1 HISTORICAL DEMOGRAPHIC DATA

Parish records of baptisms, burials, and marriages exist for the study area and are held by the Orkney County Library and Archive in Kirkwall. These records are incomplete and unsuitable for rigorous demographic analysis and have not been included in the findings described below. Beginning in 1855, civil authorities began to register vital events. The civil registers are held at the General Register Office for Scotland (GROS) in Edinburgh, which is now part of the National Records of Scotland office after merging with the National Archives of Scotland. GROS makes civil registers available to the public for an access fee through the Scotland’s People Centre. The NOPH project obtained special permission to make digital transcriptions of all of the records from northern Orkney from 1855 to 2009, the date of the last visit of a team member to the Register Office. Civil birth records contain information on the name of the child, date of birth, house or farm name and island of birth, and the names, occupations, marital status and place and date of marriage of the child’s parents. Marriage records provide information on the names and ages of the bride and groom, date and place of marriage, occupation of the bride and groom, and indicators of biological relationship between the bride and groom. Marriage records also specify the names, occupations, and marital statuses of the parents of the bride and groom in addition to whether the parents are living or dead at the time of their children’s marriage. Death records contain the name of the deceased, date of death, place of death, age at death, status, occupation of the deceased, name of the spouse of the deceased, names and occupations of the decedent’s parents, cause of death, and length of sickness. Cause of death has not yet been systematically coded using the ICD or other coding scheme (WHO, 2016).

The civil registers were transcribed as written in the original register books. After digitization, the civil records were linked to reconstruct individual life histories between 1855 and 2009. Only vital records from the six-island study area were used. Vital events could therefore not be followed if they occurred outside of the northern islands. No standardized identifying information, such as a form of numerical identification, is contained in the individual records and name redundancy is high in the northern islands, so a process of manual record linkage was used to minimize errors (Sparks, 2007). In the northern islands, certain surnames are very common. For example, 22 percent of the 1861 census population of Westray had the surname of Rendall or Drever. The range of given names is also limited, such that when taken in combination, it can be difficult to accurately assign links among a number of John Rendalls or Jane Drevers, for example. This presents a challenge for manual linkage, but it makes using an automated algorithm especially challenging. This may be a source of possible bias in data linkage. In-comers to the islands and those with unusual names will likely link at a higher rate than local Orcadians with common names if they were resident long enough to allow a sufficient number of vital events to appear in local registers. Some of the in-comers are of higher socioeconomic standing, such as doctors, clergy, and lighthouse keepers. Others, however, are itinerant laborers and traders of lower economic status. Manual linkage efforts, within the vital registers (birth to death), between censuses (1851 to 1861 and so on), and among the vital registers and censuses (birth to census(es) to death) are ongoing.

The procedure of record linkage between types of vital events began by assigning all births with a unique identifying number (UID). Birth records were then matched to marriages recorded in the study area by the bride and groom’s first names, years of birth, father’s surname, and mother’s maiden name in addition to information on the place and date of marriage if applicable. A UID was assigned to the bride or groom if each of these pieces of information matched exactly or were reasonably correct so as to account for variation in the spelling of names and slight discrepancies in year of birth. The same criteria were used to assign UIDs to death records. For each marriage, a unique family ID was generated to keep track of families (FamID). Marriages were then linked to birth records of any children produced by the couple, and the FamID was assigned to matched births. This process of nominal linkage was followed until all accurate links could be made. The majority of births occurred close to the place of marriage of the child’s parents. For example, on Sanday from 1855-2009, 81 percent of births were to a couple whose marriage was registered on Sanday or one of the other islands in the study area. On one of the smaller islands, Papa Westray, that figure is 86 percent. To date, processing and linking efforts have linked 35% of individuals in the civil registers from birth to death, although efforts to increase the number of accurate links are ongoing. The low level of linkage is primarily attributable to name redundancy and out-migration from the study area during the late 19^th^ and early 20^th^ centuries, both of which can contribute to linkage failure.

Decennial censuses began in Orkney in 1841. Individual-level data are available for all censuses up to and including 1911. The United Kingdom places a 100-year embargo on the public release of census micro-data. The 1841 census is not suitable for detailed demographic analysis as age information is rounded to 10-year intervals for adults, no relationship information within households is recorded, and little information is given for occupations. In later censuses, information is organized by household and named farmstead or house, allowing for linkage to other spatial data by farmstead name. For each household member, the census lists their name, sex, relation to household head, occupation, and from 1851-1881, the amount of land held by the house and number of laborers employed by the household head. Individuals listed in the 1851-1911 censuses are being linked across census years, allowing us to track changes in residence and household composition. The three civil registers are also being linked to individual census returns, which will allow future researchers a more complete view of individual life courses during the 1851-1911 period. Occupations from all data sources that list information on occupation are being coded using the HISCO system for future research (Van Leeuwen, Maas, & Miles, 2002).

### 3.2 HISTORICAL ECONOMIC DATA

The Lands Valuation (Scotland) Act of 1854 established a systematic valuation of land and buildings throughout Scotland. Before 1855, some valuations were recorded, but lacked consistency across locations and individual data collectors (National Archives of Scotland, 2003). Beginning in 1855, valuation rolls were recorded annually for every county in Scotland. Every house, farmstead, commercial building, or other plot of land was recorded along with information about the owner, tenant, occupier, and monetary value. Only the head of household was named in the record (as owner or tenant, for instance). Annual valuation continued until 1989, after which valuation rolls only list non-domestic properties (Cory, 2004). Valuation rolls for northern Orkney are held at the Orkney Library and Archive. Project personnel transcribed a sample of the annual evaluations, representing the years 1855, 1861, 1871, 1881, 1891, 1901, 1911, 1921, and 1931 to correspond with the years of the decennial census. The 1855 valuation was matched to the 1851 census enumeration, as it was the closest possible approximation and represented the earliest consistent valuation for the northern islands. All valuation listings from the study area were transcribed for the sampled years. The digitized valuation rolls were matched to the corresponding census enumeration by island, farm name and, where possible, the name of the household head. In the nineteenth century, some landlords owned entire islands, while other islands had more than one major landowner and several owner-occupiers. Owner-occupancy rates increased dramatically beginning in the 1920s and 1930s as large landowners began to partition and sell off their holdings, often to the tenants whose families had worked the land for generations.

It is plausible that valuation rolls reflect the amount and quality of land associated with a farmstead. To assess whether valuations generally reflect the size of holdings in both arable and pasture, and if different owners were translating acreages into valuations using different scales, we analyzed information from a sample drawn from old estate maps and documents matched to the valuation rolls (Jennings, 2010). These sources detail the amount of land in pasture and arable attached to named holdings owned by different landlords. An OLS regression predicting valuation from acres of arable, acres of pasture, and a dummy variable for landlord fit the data very well (R^2^ = 0.98; N = 50). Different landlords (N=3) did not have a significant effect on the relationship. Valuations may be used as close proxy measures for land quality and quantity in this context.

Annual agricultural prices were collected from archived records of the Orkney County Sherriff’s Court. In Scotland, each year a court convened to record the “fiars prices” of common grains. These prices reflected the prevailing prices of the crop of the preceding year, as the court met in February and recorded the prices of the harvest of the previous autumn. The harvest of autumn 1860, for example, was recorded in February 1861. These prices were often used to translate payments in kind into payments in cash and settle payments of rents and other duties. Records of rentals and payments to clergy often include the year’s fiars prices in the document. This practice was customary throughout Scotland, and can provide a consistent and comparable record of prices in the eighteenth and nineteenth centuries (Gibson & Smout, 2007; Mitchison, 1965). In Orkney, the most commonly recorded products were bere (a form of barley), malt, and oatmeal. Malt is processed barley, and the price reflects processing as well as duties, which are listed separately. Malt is the only product for which duties are mentioned in the sources. The time series of grain prices covers the late 18^th^ century the 1930s without gaps. Units of measurement and currency have been harmonized to allow for comparative analysis over time. Price data for other goods are available and have been digitized, but the coverage is incomplete. The incomplete series include poultry, butter, peat, kelp, oil, and eggs.

Additional information about the agricultural economy can be gleaned from records of reports made by the Orkney Board of Agriculture to the Scottish Government for the annual agricultural census. These agricultural census returns list details about agricultural production at the level of the parish and were collected each June. These reports are aggregates of declarations of agricultural activity made by each individual farmer to the Board of Agriculture. Government authorities did not retain the individual returns. Parishes roughly correspond to islands, but some islands, such as Sanday, have more than one parish and small islands, such as Faray, share a parish with a larger island. The parishes in the NOPH are Westray, Lady (Sanday), Cross (Sanday), Burness (Sanday), Eday and Faray, North Ronaldsay, and Papa Westray. The agricultural returns begin in 1866 and have been transcribed until 1967. The returns include the number of holdings (sometimes tabulated by holding size), total acres rented, total acres owner-occupied, acres in arable, acres in rough grazing, acres in pasture or grass, number of workers employed, and count of livestock by animal type. The format and level of detail changes over time. For example, in some years the returns specify how many acres are planted in different crops, rather than grouping all arable together. The kinds of livestock listed may vary from year to year, but always include cattle, sheep, and pigs. Poultry and horses are recorded with less consistency. Despite changes in format and kinds of information included, the agricultural returns provide an annual account of the changing composition of North Orkney’s agricultural production and land use at the parish level.

Other economic information was drawn from documents produced by the Parochial Boards of the seven parishes of the six northern islands. They include applications for poor relief and the rolls of the poor who were approved for relief. There were no workhouses in the northern islands, so all of the relief provided was “outdoor” relief, unless the recipient was committed to a hospital or asylum. The hospital was located in Kirkwall, which also served a few asylum cases, while others were sent to asylums outside of Orkney. The applications and poor rolls provide insight into which members of the community faced the greatest economic challenges. The applications and rolls provide the date of application, name, age, occupation, and marital status of the impoverished, the parish and place of residence, a justification for seeking relief, the amount of relief allowed if approved, and a justification if refused. The dates of surviving documents vary from parish to parish and these sensitive documents are sealed for 70 years, so a complete sample of a lengthy period and the complete study area is not possible. For example, Westray has the most complete coverage, from 1861 to 1930, while Lady Parish (Sanday) only has records from 1858 to 1867.

### 3.3 ETHNOGRAPHY AND ORAL HISTORY

Ethnographic interviews were conducted to complement historical documentary evidence and archaeological survey to understand the processes of change that occurred within living memory. The specific goals of the interviews were to obtain information to augment available household-level data from censuses, document changes in settlement patterns and land use, understand changes in work patterns over the last hundred years, and explore the transition from a subsistence-based, small-scale tenant farming system that relied on human and animal labor to the current large-scale owner-operated, mechanized and capital intensive system of market-oriented production that began in the 1920s and 1930s. A number of these changes can be examined through the experiences of people over the age of 60 in 2003 (born before 1943). This cohort has seen increasing nucleation of settlement during the transition from smallholding farms run by family labor and focused on subsistence to larger, mechanized farms focused on the commercial production of cattle. Orcadians over 60 have also witnessed out-migration and internal migration. With the current demographic profile in the northern islands, there are many residents within this cohort. In the 2001 census, 33 percent of the residents of the study area were over age 60, suggesting a cohort size of approximately 428. Once eligible Orkney-born residents were identified, the final cohort size was 275. One island, Faray, was abandoned in 1951, but some people interviewed on other islands once lived there and provided information for that island.

The original goal was to interview the entire cohort of native Orcadians over age 60 in the study area. The overall response rate was 87 percent (239 interviews of 275 eligible participants), which is extraordinary for ethnographic research. Interviewees were recruited through local institutions, such as the Auks’ Club (a voluntary organization of people over 60), the Scottish Women’s Rural Institute (whose members are almost all over 60), and local heritage centers. Interviewees also provided further contacts for recruitment. Radio Orkney, a program of the BBC that is aired each morning and widely listened to by Orcadians, offered to broadcast an appeal for participants, to which there was a large response. A new Care Centre opened in 2006, and project personnel visited all center residents in the targeted group. Others were recruited through public notices in shops, letters followed by telephone calls, and personal home visits. All members of the target population were contacted by one of these means, 13 percent of whom declined to participate. Project members were unable to identify any obvious traits that distinguished responders from non-responders. Some degree of self-selection is inevitable, but it is likely to be small.

Interviews included both open and close-ended questions, following protocols developed and field tested in 2004. Interviews were conducted between 2005 and 2011. The protocol began with questions about individuals’ knowledge of their family genealogy past household composition. These topics lead to questions about the work people in those households engaged in during the respondents’ childhoods and young adulthoods and the ways in which work has changed over time. Respondents were then asked about what other changes they experienced, and finally they were asked to discuss what they considered to be the greatest changes that had taken place over their lifetimes. Interviews were recorded for later transcription. The length of interviews varied from one to three hours, with most in the two-hour range. Some informants completed more than one round of interviewing, as some had more to say, but grew tired during the original interview, a common outcome within this cohort. Interviews were coded and analyzed using Ethnograph 5.07 qualitative analysis software (Seidel, 1998).

### 3.4 REMOTE SENSING

The northern islands of Orkney are virtually treeless, providing good conditions for the use of remote sensing of landscape features from satellites or aircraft as an environmental and archaeological survey method (Johnson, Sever, Madry, & Hoff, 1988; Madry & Crumley, 1990; McGovern, Sever, & Myers, 1995; Pope & Dahlin, 1989). Aerial photographs and satellite images allowed project personnel to create a complete inventory of cultural and ecological features (Stine & Decker, 1990; Wheatley & Gillings, 2002). For example, crop marks reveal subsoil structures such as old house sites, tracks, and field boundaries (Wilson, 2000), while recent satellite images can reveal coastal erosion not apparent in OSGB maps. Most importantly, satellite imagery and aerial photography allowed the project to build a natural and cultural inventory on a landscape scale (Custer, Eveleigh, Klemas, & Wells, 1986; Farley, Limp, & Lockhart, 1990; Lock & Harris, 1996), which would be nearly impossible to do by field survey alone. Historical aerial photos and historical cadastral maps when combined with high-resolution color photos allowed for longitudinal studies of household and landscape change. Evidence for past agrarian improvements that had returned to fallow could be classified and inventoried. Table 1 provides a summary of imaging, mapping, and survey sources.

### 3.5 FIELD SURVEY

In order to ground truth remote sensing and historical data from maps, project members surveyed the visible remains of abandoned farmsteads, settlements, field systems, and rural industry. Relying on a combination of photogrammetry, field survey using differential GPS, and traditional survey, we mapped all remains of dwellings and immediately associated structures. These data were later added to AutoCAD and ArcGIS to be spatially linked to digitized historical maps and remote sensing data. Detailed inventories and notes for every abandoned farmstead were collected. All interior and exterior features were accurately measured when possible. We not only documented building footprints, but also measured all remaining components of the buildings. For example, many of the buildings had intact or semi-intact slate roofs in various states of repair. Windows and doorways were components that exhibited changes relevant to use and especially changing use of buildings. Other features, such as drains in byres were documented to understand the functioning of different structures. Particular attention was paid to identifying additions or changes to architectural arrangement of buildings and potential building past use. With these data it is possible to reconstruct each building or set of buildings spatially and temporally. The surveys offer an architectural catalog, which is enhanced by geo-referencing historical cadastral maps, illustrating changes to the built environment. Conversely, field surveys provided necessary information about building and room use, not typically illustrated in historic maps.

To identify previously used field and township dykes, we relied on a mixed systematic and opportunistic approach. First, historical maps identifying previously used field dyke locations were georeferenced. Project personnel then surveyed the locations referenced in the maps buffered by a 300 meter wide survey strip, with groups of three persons walking no more than 50 meters apart, a commonly used pedestrian archaeological survey. Each person used a handheld GPS in order to provide a relative location of the dyke or dykes. The GPS units were accurate within 5 meters. Finally, coastlines were systematically surveyed for identifiable features associated with kelp production. As in the field dyke survey, teams of three people walked the coastline no more than 50 meters apart and features were recorded using handheld GPS. Importantly, features for kelp production were not fully accounted for in cadastral maps, so our field surveys provide the first full inventory of the features as they existed during NOPH field research.

### 3.6 METEOROLOGICAL OBSERVATIONS

Historical meteorological observations for Orkney are held at the Orkney Library and Archive and the Met Office Library and Archive and have been collected and digitized. The Met Office records were reports made to the Scottish Meteorological Society (Scottish Meteorological Society, 1857-1913) and the Orkney Archive holds original observation books from Deerness and Sandwick (Clouston et al., 1827-1918). Weather observations are from various locations on Mainland, Orkney, with the most complete records from Deerness and Sandwick. While these locations are not within the study area, they are the closest available historical meteorological records, with Deerness located approximately 25 km to the south. Records from the available weather stations cover the period from 1827 through 1918. Observations include total precipitation, temperature (maximum, minimum, and average), and number of days with rain. Some stations recorded information on sunshine and winds. For some stations, daily observations were recorded, while for others, only monthly figures are available.

## 4 SELECTION OF FINDINGS

### 4.1 FARM COMPLEXES

A joint analysis of census returns, archaeological surveys, and cadastral maps led to an investigation of what we call “hidden household extension.” In many of the households in the study area, several closely related families occupied adjacent houses and shared a single set of built resources such as barns, byres, stables, kilns, kitchen gardens, and other features essential for operating a small mixed-farming enterprise. Often these families were linked by a set of married brothers. Although the archaeological evidence clearly shows that these households were functioning as integrated, multiple-family farming units, census records list them as if they were autonomous households. It is in this sense that the extended households are “hidden” from the conventional historical demographer, but clearly visible in the combined archaeological and historical record. While the tracing of genealogical linkages in census records is not yet complete, we estimate that approximately 10 to 25 percent of individually listed units belong to households of this type, depending on the census year.

The NOPH team has begun to analyze the demographic and economic dynamics of these households. Sparks (2009) found evidence from the analysis of aggregate demographic data for the area that farm households and especially households who were native to the islands were able to manage their patterns of growth so to perpetuate land ownership in the mid 19^th^ to early 20^th^ centuries. Theoretical work indicates that such households may have more favorable compositions in terms of the ratio of producers to consumers than nuclear family households, yet they were fragile, rarely lasting for more than a few decades (Hammel, 2005). This prediction appears to hold in longitudinal tracking of these households through census records and archaeological evidence of changes to domestic architecture, as households are established, persist for a period of a decade or two, and then return to a simpler form. This type of household may not be unique to Orkney (see Appendix for examples). Comparable census and archaeological evidence from Shetland suggest similar arrangements (Tait, 2012). Parallels may also be drawn with household arrangements in populations elsewhere in the world, including the Mayan regions of Mesoamerica and portions of West Africa (Fortes, 1958; Murtha, 2002; Wilk, 1991). In some regions, Maya households consist of a cluster of dwellings that house closely related individuals and their families. These “household clusters” often engage in shared agricultural and other economic tasks. In West Africa, a common household form consists of several dwellings enclosed within a walled compound. The individual dwelling inhabitants may include the co-wives of polygynous marriages and their children or extended family members and their families. Indeed, it may be the case the household extension of this kind has been underestimated in contexts where researchers have relied solely on census records to reconstruct household structure and dynamics. Two example households are described in Appendix 1.

### 4.2 STANDARD OF LIVING

The majority of residents of the study area during the pre-industrial period were tenant farmers with smallholdings, fishermen, or laborers in other rural trades. With one exception from outside the study area (Rousay), Orkney did not experience the 19^th^ century Scottish Clearances, the eviction of tenant farmers from the land to open grazing for livestock (Thomson, 2000). Yet, despite escaping the hardships of the clearances, evidence suggests that for many of the islands’ 19^th^ and early 20^th^ inhabitants, the standard of living was low.

Unfavorable economic circumstances, measured by household age composition, increase the risk of death for children under age 5 in the second half of the 19^th^ century (Sparks, 2007; Sparks, Wood, & Johnson, 2013). If the number of children and elders relative to working-age adults is high, then the household may face economic strain, as the few working adults may struggle to provide for their dependents. This elevated risk persists after controlling for variability among children, islands and households. Further, twin births and 6^th^ or higher birth order children also experience elevated risk of death. These findings illustrate how intra-household competition for limited resources affects the life chances of vulnerable household members, as suggested by the resource dilution model (Donrovich, Puschmann, & Matthijs, 2014; Öberg, 2017; Riswick, 2018; Riswick & Engelen, 2018).

A growing body of literature in comparative historical demographic research posits that demographic responses to short-term economic stress are indicators of low standard of living (Bengtsson et al., 2004; Tsuya et al., 2010). Child mortality in Northern Orkney (1855-1910) was responsive to unfavorable variation in staple grain prices, particularly among the children of non-agricultural workers, who did not possess the stability of employment of tenant farmers and were obliged to purchase food at market prices (Jennings, Quaranta, & Bengtsson, 2017). In addition, poor economic conditions were associated with delayed fertility among non-agricultural workers. The fertility and mortality of the children of tenant farmers were not affected by short-term price variations, suggesting that even within the relatively flat socioeconomic structure of 19^th^ century Orkney, the households of non-agricultural workers experienced poor living standards.

### 4.3 IMPORTANCE OF THE EGG TRADE

Eggs and hens are seldom mentioned in accounts of Orcadian economic history. The ethnographic interviews conducted in the study area over several field seasons provided insight into the importance of eggs to the local economy. The words “the hens kept the house” appear in many interviews. This refers to the money women earned by raising hens and selling eggs to a processing plant in Kirkwall, the largest town in the County of Orkney. Women used the money to buy food, clothing, household necessities and some luxuries such as “sweeties” for the children. The higher education costs of many children were covered by egg money, which was said to have equaled and sometimes surpassed farm laborers’ wages. Informants explained how egg money was used to purchase the original modern machinery, such as tractors, used on farms. Some women spoke of keeping 500 or more hens at a time. Hen keeping and eggs sales contributed to the capital necessary for the increase in owner-occupancy in Orkney that began between 1921 and 1931, as locals purchased many farms from absentee landlords (Thomson, 2008a). Interviewees stated that they or their families would not have been able to buy farms or the machinery to modernize them without the money from egg sales.

### 4.4 LAND USE/LAND COVER

The commercialization of agriculture and depopulation of North Orkney have been accompanied by unique land cover changes. In the late 20^th^ century, there was an increase in smooth grassland and a decrease in arable land. Between the 1940s and 1980s, smooth grassland, which includes pasture and land used for silage, grew from 12 to 47 percent of Orkney’s land cover (Lee, 2007). Arable land decreased from 35 to 25 percent. Rough and intermediate grassland grew from 27 percent to 66 percent of land cover (Mackey, Shewry, & Tudor, 1998). These patterns are rather different than those found in mainland Scotland, which features an increase in urban land (from 3 to 4 percent of overall land cover), arable land (from 10 to 11 percent), woodland growth from 5 to 14 percent of Scotland’s land cover, and decrease to 10 from 11 percent grassland between 1947 and 1988 (Mackey et al., 1998).

Historical land use/land cover data compiled from cadastral maps provide information for the period of peak population between 1851 and 1871. Land cover in 1860 was a balanced distribution of pasture and arable. Between 1882 and 1901, significant improvement of lands and landscape change occurred as formerly marginal hillslope and marshy lands were put into use as arable. Intensification of smallholder (crofter or farmer engaged in subsistence agriculture) production focused on improving previously uncultivable land. This peak period of land use change corresponds to the beginning of population loss. Between 1901 and the 1980s, another shift in land use occurred as the area covered by arable land decreased and grassland increased. There was an increase in natural lands as the marginal areas brought into cultivation reverted back to more natural land cover. Population loss and the decrease in the number of households and farm holdings began prior to the modern shift to grassland, which likely started in the 1920s and 1930s as tenant farms became less profitable and the widespread mechanization of agriculture began.

## 5 DISCUSSION

The geographic location, historical events and trends, depopulation, and record availability of the northern Orkney Islands in combination with the multidisciplinary approach of the NOPH allow for the joint analysis of population, environmental, and economic change over a period that spans several generations. While time, labor, and data-intensive, the design and methodology employed by this project offers insight that would not be possible if the individual arms of the study were conducted in isolation. This approach mirrors the principles of triangulation and complementarity of mixed-methods research (Greene, Caracelli, & Graham, 1989). Quantitative and qualitative approaches from multiple disciplines are combined to address a set of research questions from more than one perspective, to aid in inference and interpretation, and to capitalize on methodological strengths while attempting to counterbalance potential shortcomings or biases of a single approach (Mark & Shotland, 1987).

Linking micro-demographic information on household composition and change to studies of household architecture and landscape analysis offers potentially transformative understandings of how physical remains of households are modified and reflect those patterns and how household dynamics influence long-term regional landscape change. For example, while general patterns of land cover change in North Orkney contrast the trends for mainland Scotland, we can track why these patterns are different in Orkney because of the information derived from historical cadastral maps. Marginal landscapes were improved to meet the needs of a demographically saturated landscape. As population loss quickened, these now improved landscapes were abandoned. Linking these diverse data sets provides a rich landscape narrative to understand the relationships between households and land use decision-making. Such narratives can also be observed for individual households, often illustrating how the changing patterns of use for buildings or gardens reflected changing dynamics of household labor availability and resource needs. In archaeology, we commonly only observe the final remains of households and struggle to disentangle the palimpsest. Archaeological landscapes are no different. The micro-demographic studies became a foundational element to strengthen anthropological interpretations of households and landscapes. Uniquely, it rehumanizes land use change narratives, which commonly describe how landscapes change but rarely can illustrate how they change.

From a historical demographic perspective, linking micro-demographic data to archaeological and landscape data allow for analyses of living arrangements and the spatial distribution of people that provide detail beyond that offered by census returns and vital records. Household structure, formation, and composition have long been topics of interest to historical demographers of Europe (Laslett & Wall, 1974; Laslett, Robin, & Wall, 1983), and remain a topic of theoretical debate and empirical study (Carmichael, de Pleijt, van Zanden, & De Moor, 2016; Dennison & Ogilvie, 2014; Engelen & Wolf, 2005; Gruber & Szołtysek, 2012; Puschmann & Solli, 2014; Ruggles, 2009; 2010; Szottysek & Gruber, 2014; Szołtysek, Gruber, Zuber-Goldstein, & Scholz, 2011). The dataset produced by this study is able to parse aspects of family, household, domestic architecture, and land in a way that is impossible in population reconstructions that rely only on documentary evidence. While single community studies are inherently limited in scope and generalizability, the information gleaned from multiple data sources should motivate an evaluation of the ways that researchers have interpreted historical demographic documents, especially the assumptions that link census listings to living arrangements and the social and economic functions of the household. Further, comparative research using price data to assess living standards has demonstrated one way that multiple sources of data, in this case micro-demographic and economic data, can provide insight to variation in living standards both within and between societies (Bengtsson, Campbell, & Lee, 2004; Jennings, Quaranta, & Bengtsson, 2017; Lundh & Kurosu, 2014; Tsuya et al., 2010).

The complex interactions among households, demography, land use, and economy are often hidden by targeted research that addresses specific questions about historic processes. For example, studying the population change in Orkney by relying only on census records would have obscured many of the complex interactions that accompanied secular trends in fertility, mortality, and population size. Rich narratives of landscape, household, and family are beginning to emerge even with preliminary analyses of these data. No doubt, such data are complex and challenging to collect, maintain, and analyze. With a deep, context-driven examination of a geographically bounded area, we were able to manage some of the challenges and begin to document and understand the deeper history of Orkney’s population and how deeply this history is embedded in the broader landscape.

The research model laid out by the NOPH aims to place demographic, economic, and ecological change in context. Efforts to compile large-scale comparative data across broad regions are laudable, but this does not diminish the value of detailed mixed-methods approaches. While this study design may not be feasible everywhere in Europe or further afield, as data preservation and availability varies, we encourage researchers to consider broadening the scope of their data collection plans whenever possible to include sources that can provide the detail required to place people in the physical and built environment to improve our understanding of the processes of change.

As a case study, this project can provide insight into the demographic challenges of population aging and rural population loss. These processes began early in Orkney (mid-19^th^ century) and continue to this day. Over the study period, Orkney experienced agricultural intensification to the limits of arable production, then extensification and the shift to poultry and livestock production. Later, poultry production declined, and livestock production became increasingly intensive. In recent years, the islanders have looked to the energy and tourism sectors as new sources of support. In interviews, many native Orcadians expressed concern about the loss of young adults from the islands, as this group typically seeks higher education and work in urban areas. Others noted concerns about maintaining services for a decreasing population, especially schools. School closures on the outlying islands are commonly described as sad events that forestall the waning of local communities. These processes began before strong institutions and social services for elders were available. Research on this case of adaptation to population loss and aging may inform other contexts where populations will grow old before there are adequate non-family provisions for older adults. Further, other European regions may look to Orkney as a bellwether for similar processes of rural population change that are now underway.

## Figures and Tables

**Table 1:** Summary of map, image, and survey sources with date(s) of production

Source	Year
Archaeological Surveys	2003-2010
IKONOS 1m Satellite Imagery	2000-2015
1:250000 Soil Maps	1982
Color Aerial Photos	1983
OSGB Map	1972
WWII B/W Aerial Photos	c. 1940
1:2500 OSGB Map	1901
1:2500 Color OSGB Map	1882
Estate Reorganization Maps	1850-1880
Estate Maps	1747-1850
Modern OSGB Maps	2000-2010

**Figure A1:**
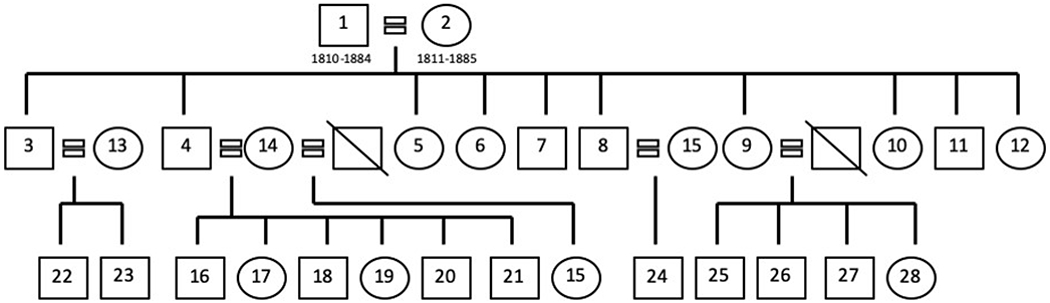
Geneology of the inhabitants of Farmstead A

**Figure A2:**
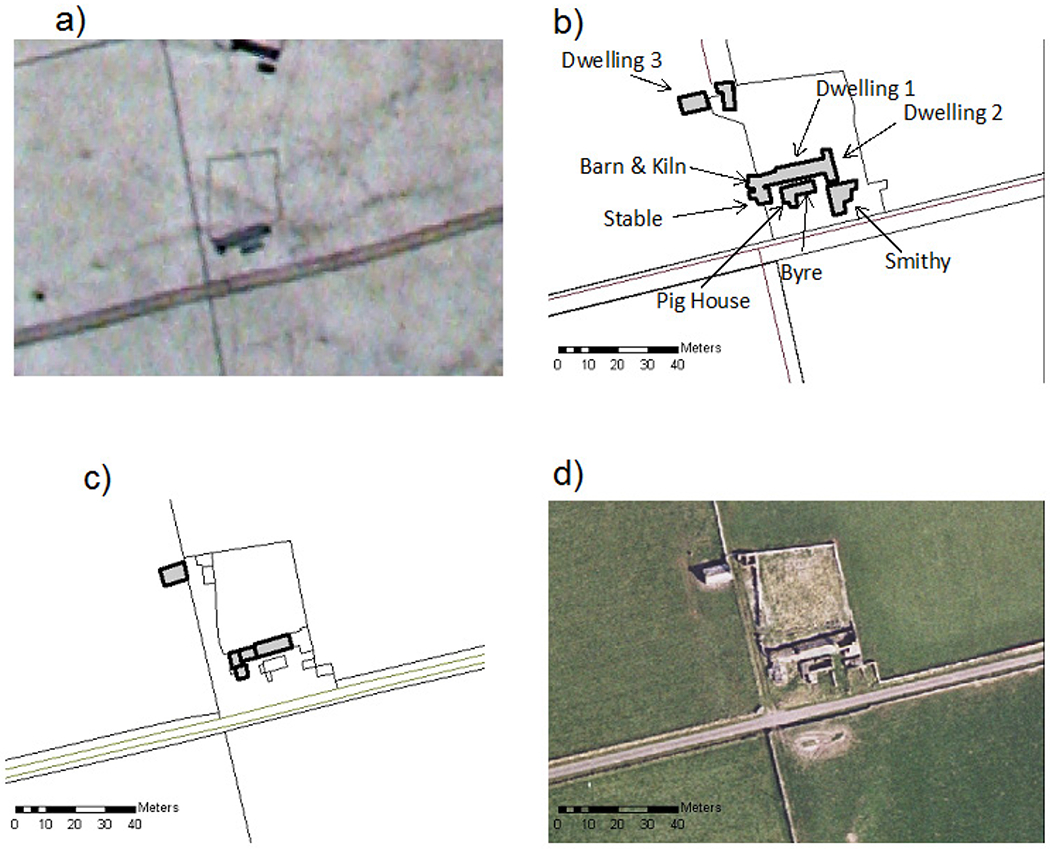
Maps and aerial photo of Farmstead A: a) pre-1850 estate map b) 1901 OSGB c) modern OSGB d) modern aerial photo. Structures identified by archaeological survey

**Figure A3:**
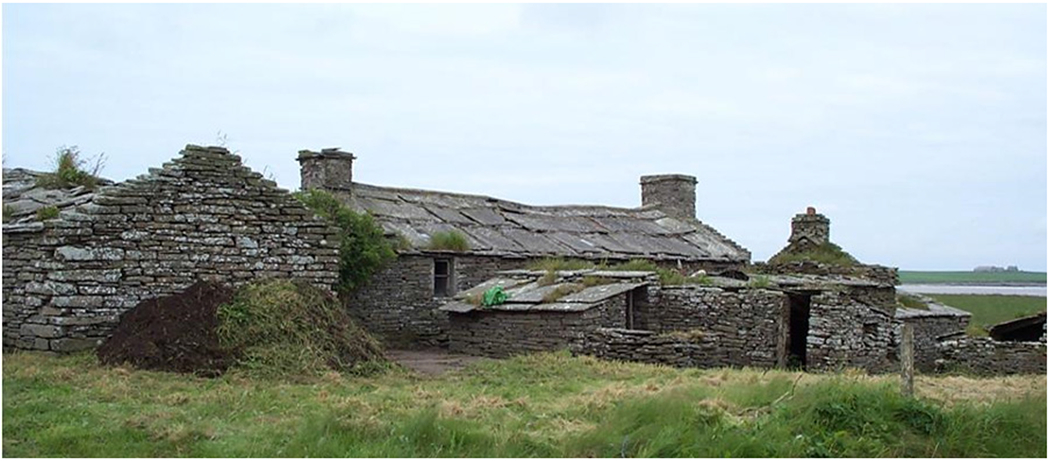
Farmstead A in 2005. Photograph courtesy of Jim Wood

**Figure A4:**
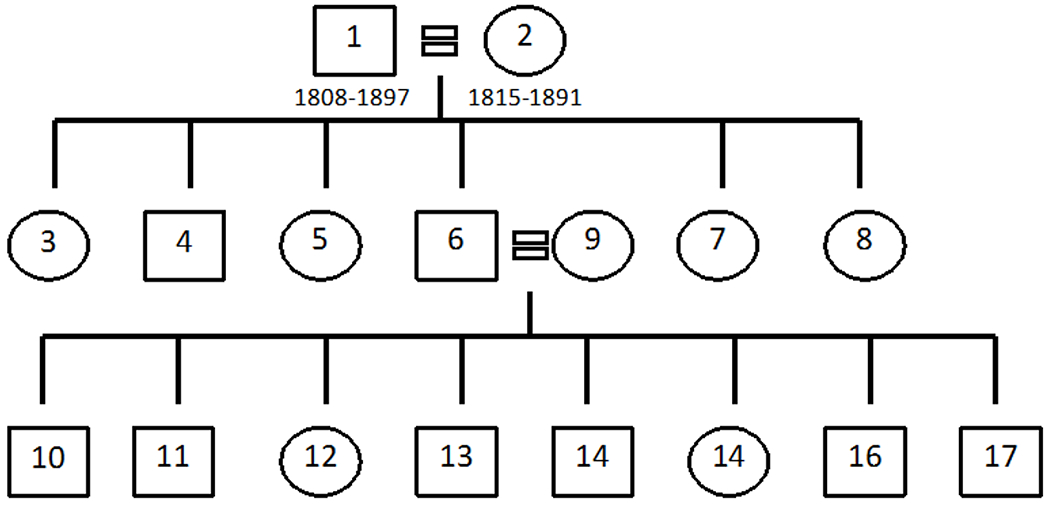
Genealogy of the inhabitants of Farmstead B

**Figure A5:**
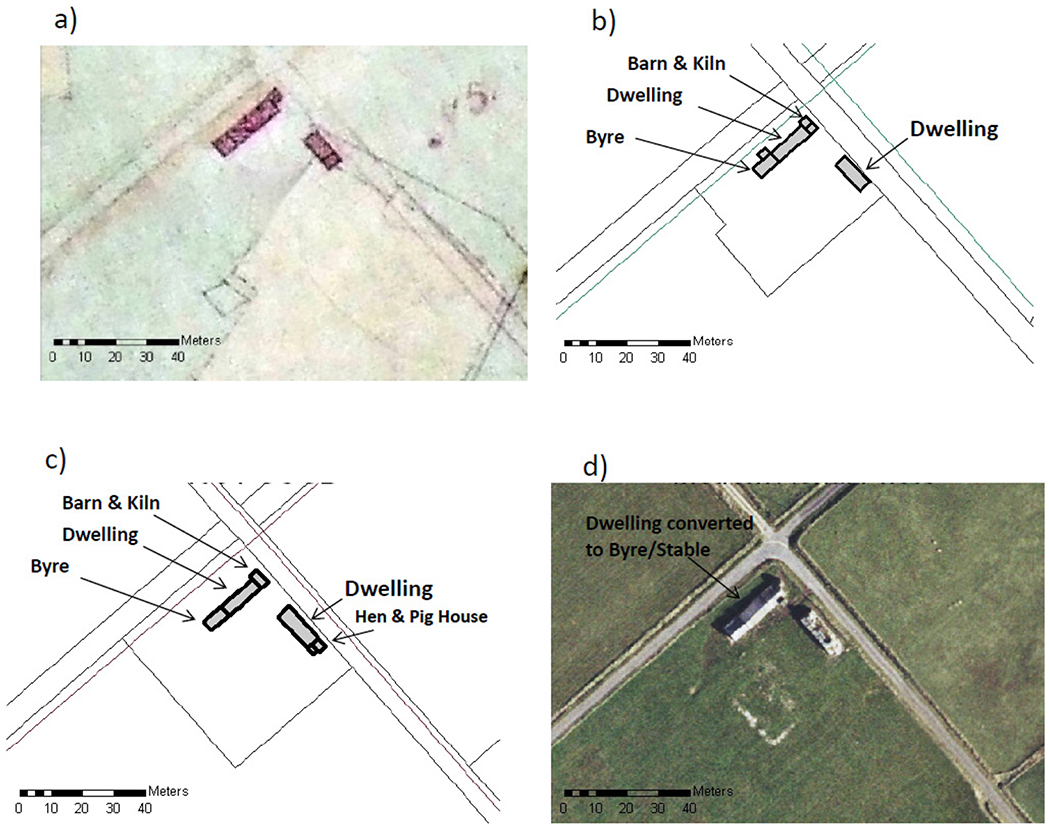
Maps and aerial photo of Farmstead B: a) 1848 estate map b)1882 OSGB c)1901 OSGB d) modern aerial photo. Structures identified by archaeological survey

**Figure A6:**
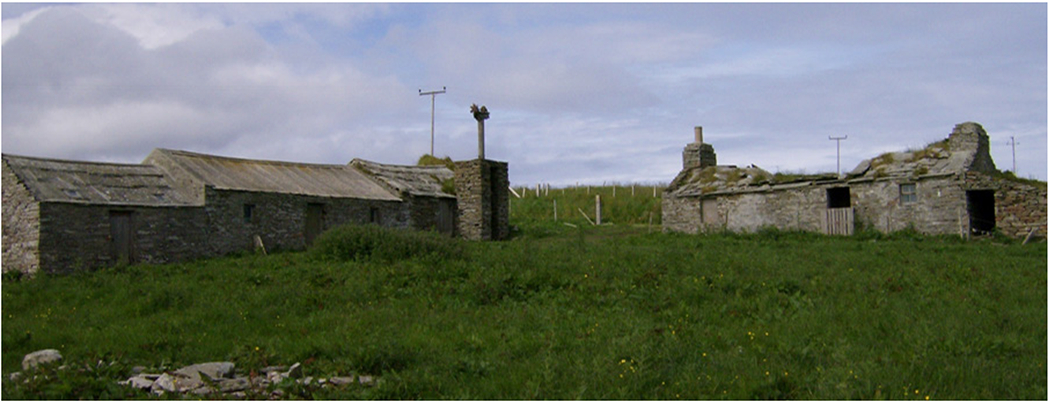
Farmstead B in 2007. Photograph by Julia Jennings

## APPENDIX

### Examples of joint analysis of household structure and dynamics using documentary and archaeological evidence

These example farmsteads from the study area illustrate the interdisciplinary analysis of household structure, living arrangements and change over time (Jennings, 2010). To describe these households over the period from 1851-1901, we use a range of data. Census returns list the household inhabitants and vital registers are used to verify and supplement the genealogical relationships among household members. Historical maps, modern aerial photography, and archaeological survey identify domestic and agricultural structures and architectural change over time. Ethnographic interviews provide additional verification and detail.

#### “Farmstead A”

Farmstead A was a 10-acre croft. The inhabitants in the census years 1851-1901 are shown in Table A1. Figure A1 illustrates the genealogical relationships among these individuals in the 50-year interval. At its height, the farm compound consisted of three dwellings, two of which, along with a barn and a grain-drying kiln, were arranged in a single linear block (Figure A2). A flagstone pathway (close) separates this block from the byre, stable, and pig houses. A cluster of buildings associated with a smithy is also separated from the main block. A third dwelling was constructed after (and somewhat further from) the other buildings at the northwest corner of the kailyard (house garden enclosure).

In 1851, Farmstead A is occupied by a farmer, Individual 1, his wife Individual 2, and their children (individual ID numbers correspond to those of Table A1 and Figure A1). At this time, Farmstead A is a simple family household. In 1861, their second son, Individual 4, has become a blacksmith. The smithy buildings may date to near this time. By 1871, changes have come to the croft. It is now a compound household, headed by Individual 1 and his sons 4 and 8, and their wives and children. 1 is still listed as the farmer of Farmstead A, while 4 and 8 are blacksmiths. Given this change in family structure, the additional dwelling to the north of the main house probably dates to this time. The easternmost dwelling may also represent an addition to accommodate new household units.

In 1881, the household retains much of the same configuration, but now 8 is listed as a fisherman. The Ordinance Survey map from 1882, in combination with archaeological surveys of the remaining buildings demonstrate that each family unit likely occupied a separate dwelling, while sharing a single set of outbuildings, including the barn, kiln, byre, stable, pig houses, and smithy buildings (Figure A2). By the census of 1891, 1 and 2 have died, and 3, their eldest son, has returned to Farmstead A with his family. Now, the heads of the three units of the compound household are all brothers and 3 is now identified as the farmer. In 1901, 4 and his family have left Farmstead A, and 9, now a widow, has returned to her childhood home with her children. Oral histories indicate that 9 and her children occupied the northern dwelling, 3 and his family occupied the western dwelling in the main block, and 8 and his family occupied the eastern dwelling.

Farmstead A represents the most detailed reconstruction of a farmstead and its history that is possible with current data sources. One of the dwellings was occupied until 1989, and the buildings remain remarkably well preserved and are now under the care of a local Buildings Preservation Trust, which had restored and preserved this traditional farmstead (see Figure A3 for a modern photo of the croft complex). This has allowed for thorough archaeological study combined with information gathered from historical records and the neighbors and relatives of the last living inhabitant, who died in 1993. Farmstead A was the starting point for much of our work with the interdisciplinary study of households, as it first revealed that households listed in the censuses with separate heads (but with a shared farm name, sometimes distinguished by number) could be occupied by a single economically interdependent group of relatives.

**Table A1:** Census listings of the inhabitants of Farmstead A

	Genealogy Number	Relationship to Head	Age	Year of Birth	Occupation
**Inhabitants of Farmstead A, 1851**					
Farmstead A	6	Daughter	6	1845	
Farmstead A	2	Wife	40	1811	
Farmstead A	1	Head	41	1810	Farmer (10 acres employs 2 laborers)
Farmstead A	7	Son	4	1847	
Farmstead A	9	Daughter	1	1850	
Farmstead A	5	Daughter	10	1841	
Farmstead A	4	Son	14	1837	
Farmstead A	8	Son	2	1849	
					
**Inhabitants of Farmstead A, 1861**					
Farmstead A	6	Daughter	16	1845	
Farmstead A	2	Wife	50	1811	
Farmstead A	1	Head	51	1810	Farmer
Farmstead A	7	Son	14	1847	
Farmstead A	11	Son	8	1853	
Farmstead A	12	Daughter	4	1857	
Farmstead A	10	Daughter	10	1851	Scholar
Farmstead A	5	Daughter	20	1841	
Farmstead A	4	Son	24	1837	Blacksmith
Farmstead A	8	Son	12	1849	Scholar
					
**Inhabitants of Farmstead A, 1871**					
Farmstead A 1	15	Step-Daughter	11	1860	Scholar
Farmstead A 1	16	Son	6	1865	Scholar
Farmstead A 1	17	Daughter	3	1868	
Farmstead A 1	18	Son	2	1869	
Farmstead A 1	12	Wife	38	1833	
Farmstead A 1	4	Head	36	1835	Blacksmith
					
Farmstead A 2	2	Wife	60	1811	
Farmstead A 2	1	Head	61	1810	Farmer
Farmstead A 2	9	Daughter	21	1850	
Farmstead A 2	12	Daughter	14	1857	
					
Farmstead A 3	15	Wife	27	1844	
Farmstead A 3	8	Head	22	1849	Blacksmith
					
**Inhabitants of Farmstead A, 1881**					
Farmstead A 1	2	Wife	70	1811	
Farmstead A 1	1	Head	71	1810	Farmer
Farmstead A 1	12	Daughter	23	1858	General domestic servant
					
Farmstead A 2	19	Daughter	9	1872	Scholar
Farmstead A 2	16	Son	16	1865	Blacksmith Apprentice
Farmstead A 2	18	Son	12	1869	Scholar
Farmstead A 2	14	Wife	48	1833	
Farmstead A 2	20	Son	6	1875	Scholar
Farmstead A 2	4	Head	44	1837	Master Blacksmith
Farmstead A 2	21	Son	4	1877	
					
Farmstead A 3	15	Wife	37	1844	
Farmstead A 3	24	Son	9	1872	
Farmstead A 3	8	Head	32	1849	Fisherman
**Inhabitants of Farmstead A, 1891**					
Farmstead A 1	15	Wife	47	1844	
Farmstead A 1	24	Son	19	1872	Fisherman
Farmstead A 1	8	Head	42	1849	Fisherman
Farmstead A 1	N/A	Boarder	7	1884	Scholar
					
Farmstead A 2	14	Wife	57	1834	
Farmstead A 2	4	Head	53	1838	Blacksmith
Farmstead A 2	21	Son	14	1877	Blacksmith Apprentice
					
Farmstead A 3	22	Son	22	1869	Farm Servant
Farmstead A 3	13	Wife	48	1843	
Farmstead A 3	3	Head	56	1835	Farmer
Farmstead A 3	23	Son	15	1876	Farm Servant
					
**Inhabitants of Farmstead A, 1901**					
Farmstead A 1	27	Son	13	1888	Scholar
Farmstead A 1	9	Head	51	1850	Housekeeper
Farmstead A 1	28	Daughter	11	1890	Scholar
Farmstead A 1	26	Son	17	1884	Ploughman on Farm
Farmstead A 1	25	Son	21	1880	Ploughman on Farm
					
Farmstead A 2	13	Wife	58	1843	
Farmstead A 2	3	Head	66	1835	Farmer
Farmstead A 2	23	Son	24	1877	Assisting on Farm
					
Farmstead A 3	15	Wife	56	1845	
Farmstead A 3	24	Son	29	1872	Fisherman
Farmstead A 3	8	Head	52	1849	Fisherman

#### Farmstead B

Farmstead B provides an example of how households repurposed the physical structures of their farmstead to accommodate changes in household size and composition (Table A2, Figure A4). Farmstead B was a croft of 15 acres, consisting of two blocks of buildings perpendicular to each other. One block contains a dwelling, hen house, and pig house. The other block features a dwelling, barn, kiln, and byre. The order of construction of the blocks is not known, but the farm dates to at least 1848, when both blocks were included in a map of a large estate (Figure A5). Although now abandoned, this farmstead was occupied until the modern period, and some of its former inhabitants are still living and were interviewed by project personnel. This relatively recent occupation has contributed to the excellent preservation of the buildings, which have been subject to archaeological survey.

In 1851, Individual 1 and his family lived at Farmstead B. 1 was a farmer and head of a simple-family household. The household remained largely unchanged until 1871, when 1’s youngest son (6) is listed as a fisherman. In 1881, Farmstead B became a compound household, with 1 heading one unit, and 6, now married with children, heading the other. Individual 1 is still the farmer of Farmstead B, while his son 6 is a sailor in the merchant service (one of the few available fulltime employments). In 1891, the household arrangements are largely the same, but now 6 is also listed as a farmer. He has presumably returned from sailing to help work the farm, as his father is now 83 years old. His mother had died shortly after the 1891 census enumeration, and his father would pass away in 1897. Thus, by 1901, the household has returned to a simple-family household.

Unlike at Farmstead A, it is not possible to determine which nuclear families resided in which dwellings at Farmstead B. Between 1871 and 1881, one of the blocks may have been repurposed to accommodate the formation of a compound household, as both blocks were present since 1848. In 1881 and 1891, Farmstead B was a compound household, and the two dwelling units shared a single set of farm buildings. Later (the exact timing is unclear, but presumably post-1891), the dwelling in the western block was repurposed (perhaps for the second time) into a byre, and the fireplaces were closed off and stalls and muck troughs were added to accommodate cattle (Figure A5 and Figure A6 for a modern photograph).

**Table A2:** Census listings of the inhabitants of Farmstead B

	Genealogy Number	Relationship to Head	Age	Year of Birth	Occupation
**Inhabitants of Farmstead B, 1851**					
Farmstead B	3	Daughter	11	1840	Scholar
Farmstead B	2	Wife	36	1815	
Farmstead B	4	Son	7	1844	Scholar
Farmstead B	5	Daughter	5	1846	Scholar
Farmstead B	1	Head	43	1808	Farmer (15 acres)
Farmstead B	6	Son	3	1848	
					
**Inhabitants of Farmstead B, 1861**					
Farmstead B	3	Daughter	21	1840	
Farmstead B	2	Wife	45	1816	
Farmstead B	7	Daughter	8	1853	
Farmstead B	8	Daughter	2	1859	
Farmstead B	1	Head	53	1808	Farmer
Farmstead B	6	Son	13	1848	
					
**Inhabitants of Farmstead B, 1871**					
Farmstead B	2	Wife	56	1815	
Farmstead B	7	Daughter	18	1853	
Farmstead B	8	Daughter	12	1859	
Farmstead B	1	Head	63	1808	Farmer
Farmstead B	6	Son	23	1848	Fisherman
					
**Inhabitants of Farmstead B, 1881**					
Farmstead B No1	2	Wife	66	1815	
Farmstead B No1	7	Daughter	28	1853	
Farmstead B No1	8	Daughter	22	1859	
Farmstead B No1	1	Head	73	1808	Farmer
					
Farmstead B No2	9	Wife	29	1852	
Farmstead B No2	10	Son	6	1875	Scholar
Farmstead B No2	12	Daughter	2	1879	
Farmstead B No2	6	Head	33	1848	Sailor, merchant service
Farmstead B No2	11	Son	4	1877	
					
**Inhabitants of Farmstead B, 1891**					
Farmstead B No1	9	Wife	38	1853	
Farmstead B No1	12	Daughter	12	1879	
Farmstead B No1	15	Daughter	2	1889	
Farmstead B No1	14	Son	3	1888	
Farmstead B No1	13	Son	8	1883	(Dumb)
Farmstead B No1	6	Head	43	1848	Farmer
					
Farmstead B No2	2	Wife	76	1815	
Farmstead B No2	8	Daughter	32	1859	
Farmstead B No2	1	Head	83	1808	Farmer
					
**Inhabitants of Farmstead B, 1901**					
Farmstead B	14	Daughter	12	1889	Scholar
Farmstead B	9	Wife	49	1852	
Farmstead B	16	Son	9	1892	Scholar
Farmstead B	17	Son	7	1894	Scholar
Farmstead B	6	Head	53	1848	Farmer, Horseman
